# Supplementary material for: PTH induces bone loss via microbial-dependent expansion of intestinal TNF+ T cells and Th17 cells
Source: Nat Commun. 2020 Jan 24;11:468. doi: 10.1038/s41467-019-14148-4 (PMC6981196; doi:10.1038/s41467-019-14148-4)
Supplement: Supplementary file 1 — Supplementary Information [file 41467_2019_14148_MOESM1_ESM.pdf]

PTH INDUCES BONE LOSS VIA MICROBIAL-DEPENDENT EXPANSION OF INTESTINAL TNF⁺ T CELLS AND TH17 CELLS

Mingcan Yu eat al

Supplemental figures

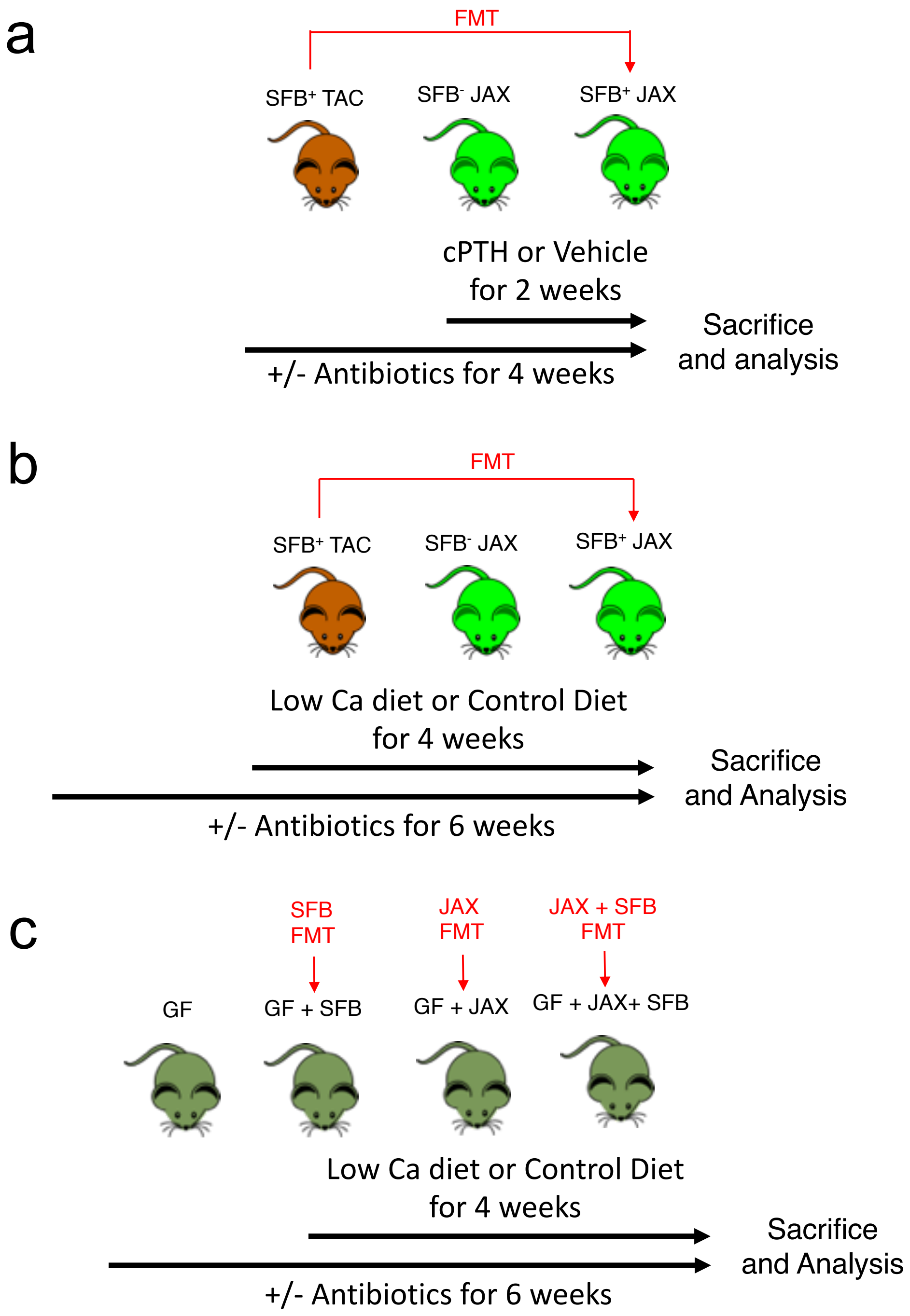

Supplemental Figure 1. Diagrammatic representation of the experimental models. a. Continuous PTH (cPTH) treatment as a model of primary hyperparathyroidisn. SFB$^+$ JAX mice were generated by fecal microbiome transfer (FMT) that involves oral gavaging SFB$^-$ JAX mice with a liquid suspension of fecal pellets collected from SFB$^+$ TAC mice. **b**. Low calcium diet as a model of secondary hyperparathyroidisn. SFB$^+$ JAX mice were generated by fecal microbiome transfer (FMT) that involves oral gavaging SFB$^-$ JAX mice with a liquid suspension of fecal pellets collected from SFB$^+$ TAC mice. **c**. Fecal microbiome transfer (FMT) as a model to investigate the specific role of SFB. Germ-free (GF) mice were subjected to FMTs to generate mice colonized exclusively with SFB. Controls included GF mice colonized with the SFB$^-$ JAX microbiota and mice colonized with both SFB and the SFB$^-$ JAX microbiota. The figure contains elements from Clipart Library (http://clipart-library.com).

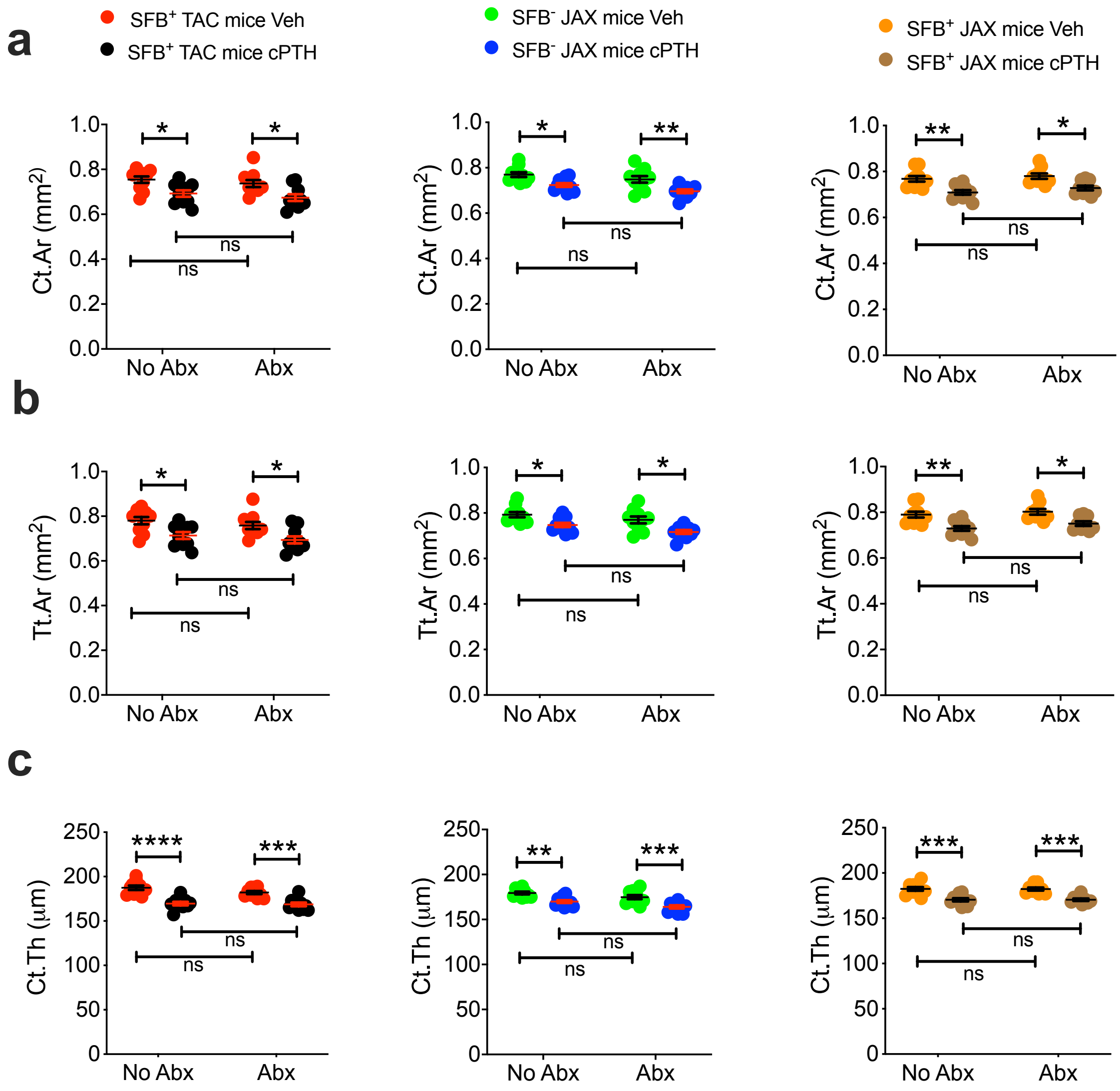

Supplemental Figure 2. The microbiota is not required for cPTH treatment to alter cortical structure. Cross-sectional measurements of femoral cortical structure by in vitro µCT scanning. SFB$^+$ and SFB$^-$ TAC and JAX mice were treated with vehicle or cPTH for 2 weeks and with antibiotics (Abx) or without antibiotics (No Abx). **a.** Cortical bone area (Ct.Ar), n= 9-11 mice per group. **b.** Total cross-sectional area inside the periosteal envelope (Tt.Ar), n= 9-11 mice per group. **c.** Average cortical thickness (Ct.Th), n= 9-11 mice per group. Data are expressed as Mean ± SEM. All data were normally distributed according to the Shapiro-Wilk normality test and analyzed by two-way analysis-of-variance and post hoc tests applying the Bonferroni correction for multiple comparisons. *=p<0.05, **=p<0.01, ***=p<0.001 and ****= p<0.0001 compared to the indicated group. Source data are provided as a Source Data file.

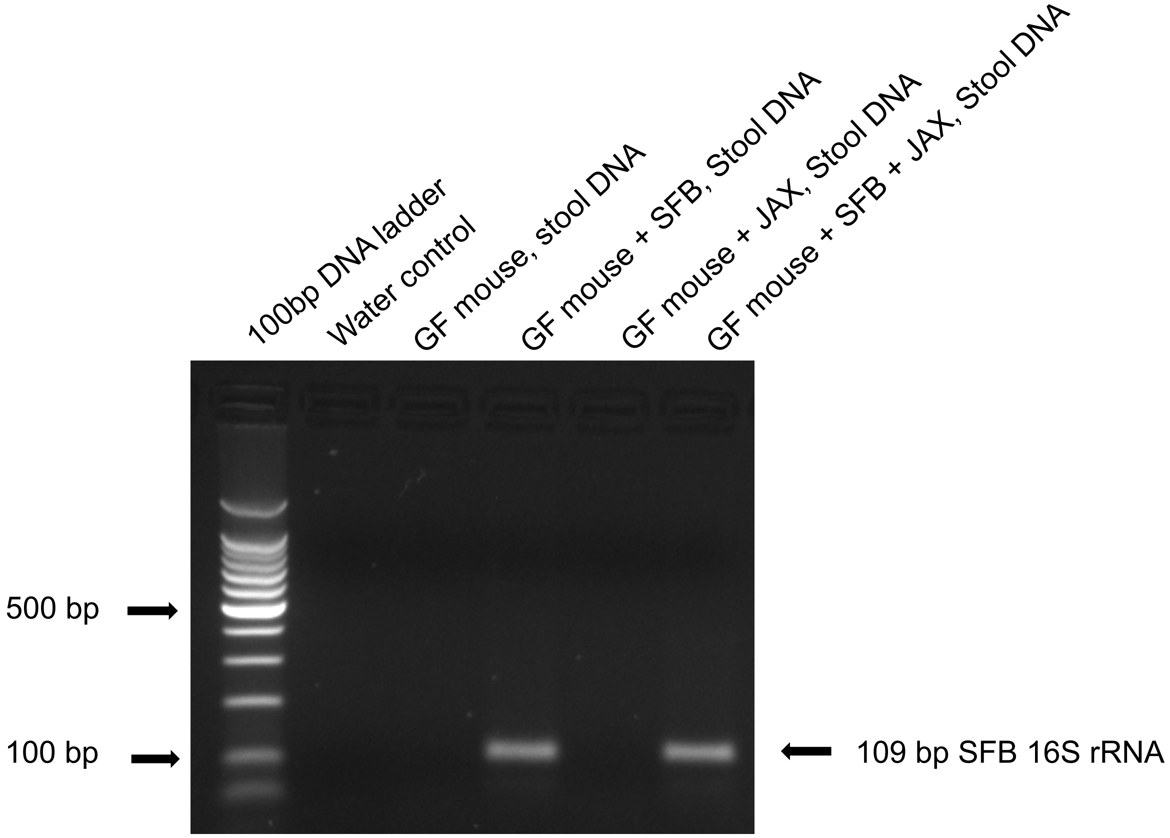

Supplemental Figure 3. Detection of Segmented filamentous bacteria (SFB) in C57BL/6 mice. Detection of SFB in fecal pellets collected from the indicated C57BL/6 mice using SFB-specific primers. PCR amplification was done using DNA purified from mouse pellets as template, and amplicons detected by electrophoresis using a 2% agarose gel. Fecal samples were collected from Germ-free (GF) mice, GF mice mono-colonized with SFB, GF colonized with SFB^- JAX by fecal microbiome transplant (FMT), and GF mouse colonized with SFB^- JAX by FMT and with SFB. Primers used for PCR are described in Supplemental Table 1.

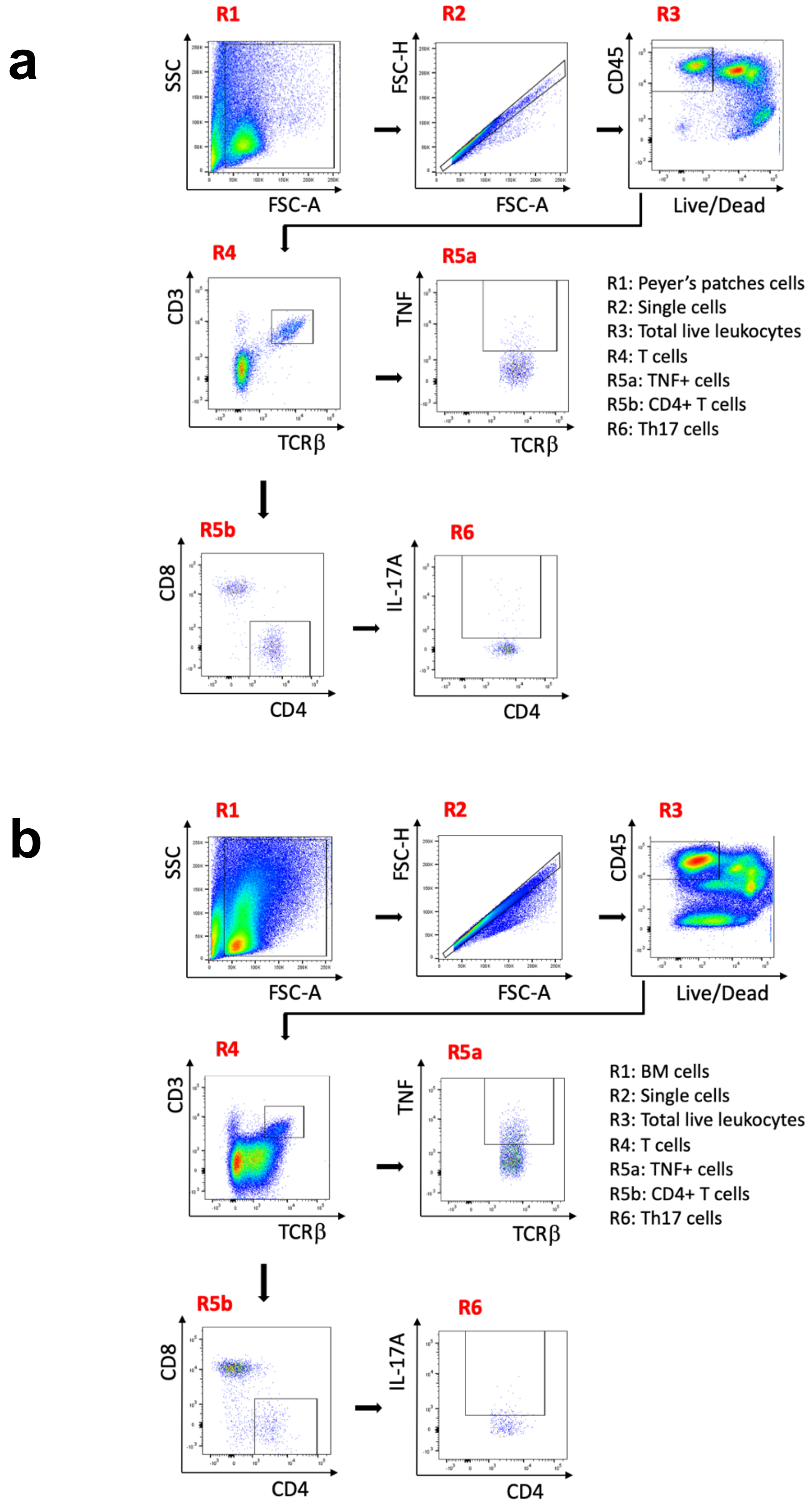

Supplemental Figure 4. **Gating strategy used to identify Payer's patches (Panel a) and BM (Panel b) TNF^{+} T cells and Th17 cells**. Following red blood cells lysis, single cell suspensions were prepared from Payer's patches and BM and stained with antibodies to the indicated antigens and live/dead cell dye. Gated regions are numbered from R1 to R6. The figure shows one representative gating of flow cytometric plot.

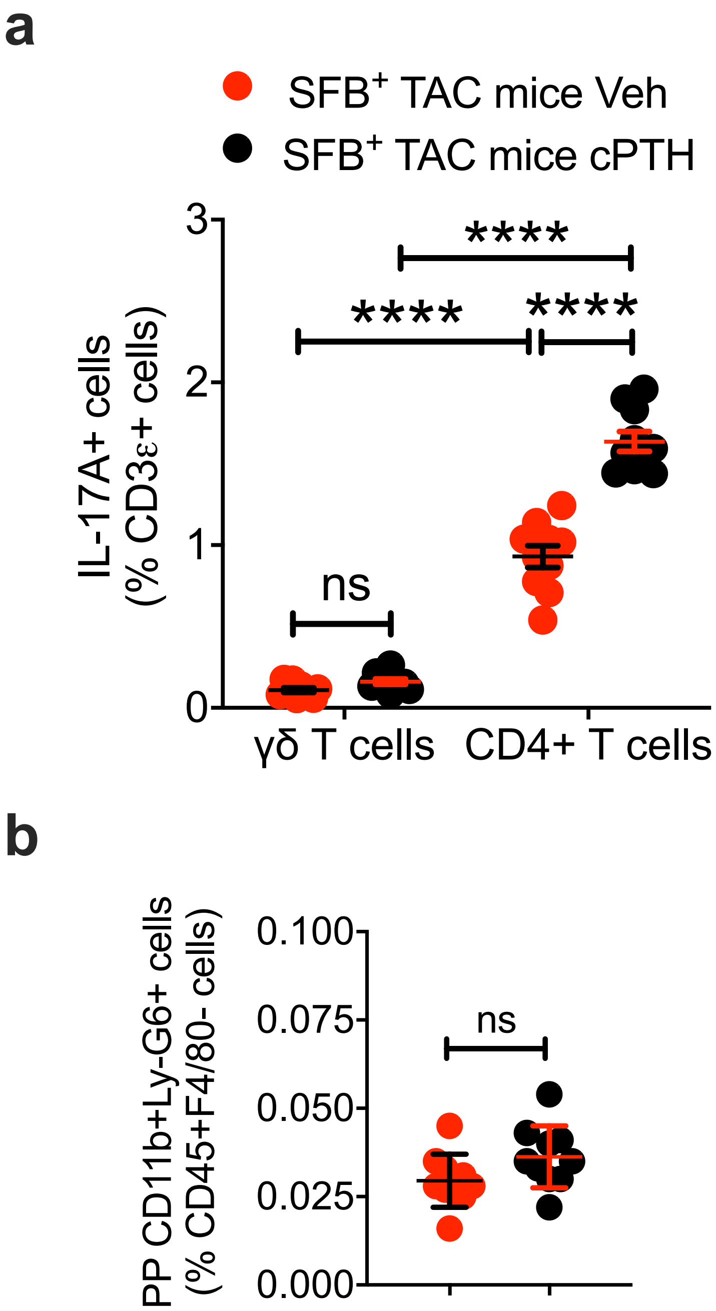

Supplemental Figure 5. Effects of cPTH treatment on the relative frequency of PP IL-17A^{+} γδ T cells, Th17 cells and neutrophils. **a**. Relative frequency of PP IL-17A^{+} γδ T cells and PP IL-17A^{+} CD4^{+} cells expressed as percentage of total T cells (CD3ε^{+} cells). **b**. Relative frequency of PP neutrophils. SFB^{+} TAC mice were treated with cPTH for 2 weeks. n= 10 mice per group. Data are expressed as Mean ± SEM. All data were normally distributed according to the Shapiro-Wilk normality test. Data in panel a were analyzed by two-way analysis-of-variance and post hoc tests applying the Bonferroni correction for multiple comparisons. Data in panel b were analyzed by unpaired t-tests. ****= p<0.0001 compared to the indicated group. Source data are provided as a Source Data file.

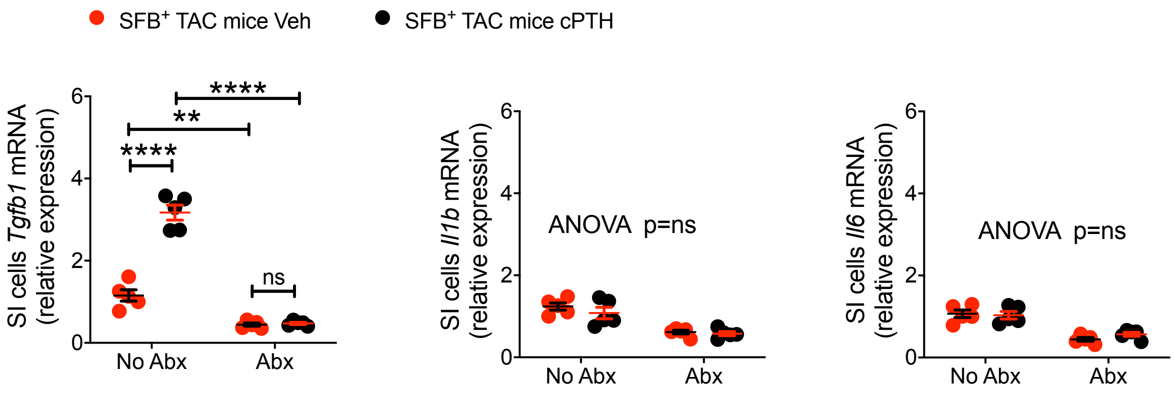

Supplemental Figure 6. cPTH treatment increases SI cells mRNA expression of *TGFβ1*, but not that of *IL-1* and *IL-6*. SFB^{+} TAC mice treated with cPTH for 2 weeks and with antibiotics (Abx) or without antibiotics (No Abx). n= 5 mice per group. Data are expressed as Mean ± SEM. All data were normally distributed according to the Shapiro-Wilk normality test and analyzed by two-way analysis-of-variance and post hoc tests applying the Bonferroni correction for multiple comparisons. **=p<0.01 and ****= p<0.0001 compared to the indicated group. Source data are provided as a Source Data file.

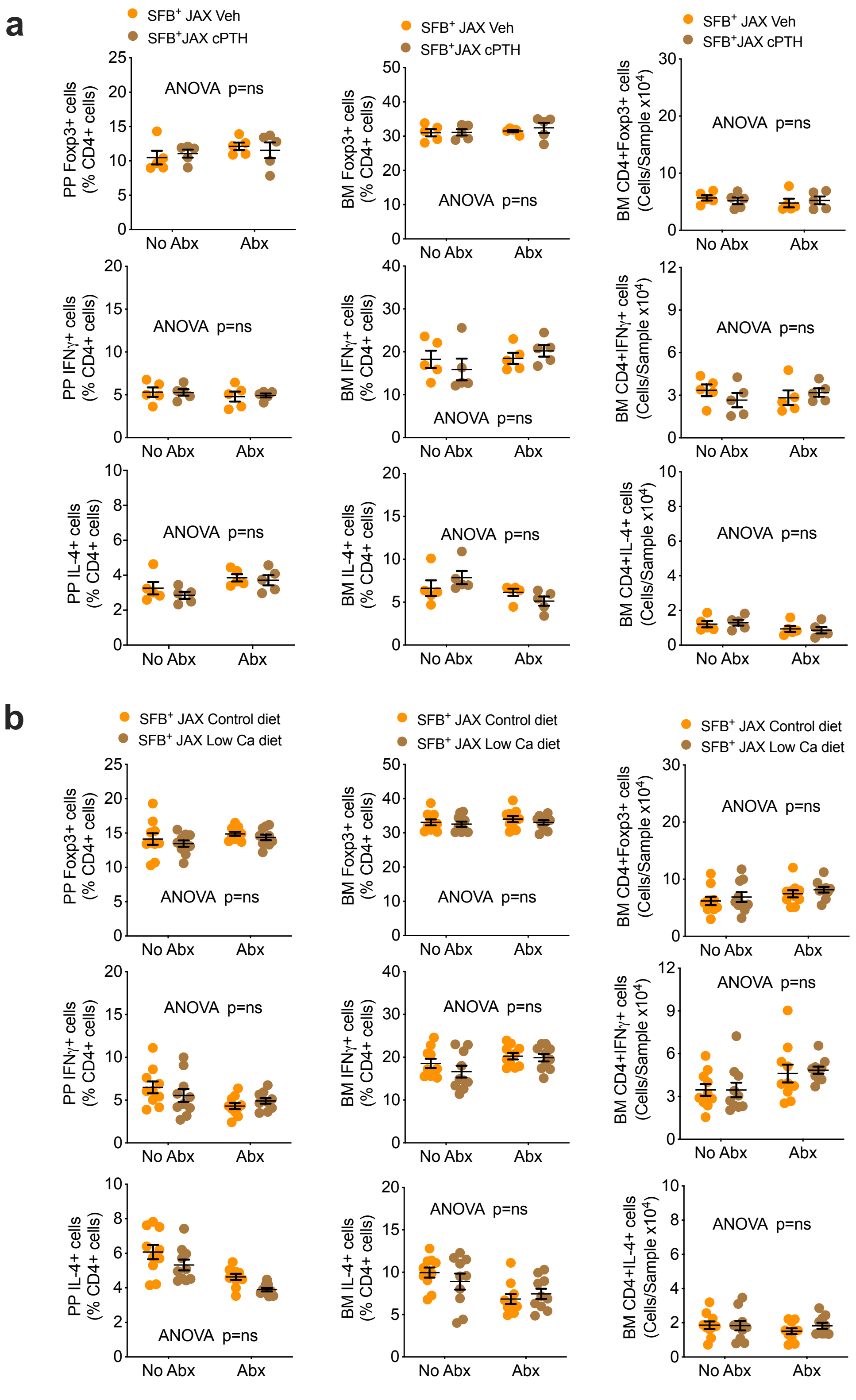

Supplemental Figure 7. **cPTH and low calcium diet do not affect the frequency of PP and BM Foxp3+ CD4+ T cells, IFNγ+ CD4+ T cells and IL-4+ CD4+ T cells. cPTH and Low calcium diet did not affect the frequency of PP and BM Foxp3+ T cells, IFNγ+ T cells and IL-4+ T cells. a.** PP and BM Foxp3+ T cells, PP and BM IFNγ+ T cells and PP and BM IL-4+ T cells in SFB+ JAX mice treated with vehicle or cPTH for 2 weeks, n= 5 mice per group. **b.** PP and BM Foxp3+ T cells, PP and BM IFNγ+ T cells and PP and BM IL-4+ T cells in SFB+ JAX mice treated with control diet or low calcium diet for 4 weeks. All animals were treated with antibiotics (Abx) or without antibiotics (No Abx), n= 10 mice per group. Data are expressed as Mean ± SEM. All data were normally distributed according to the Shapiro-Wilk normality test and analyzed by two-way analysis-of-variance and post hoc tests applying the Bonferroni correction for multiple comparisons. Source data are provided as a Source Data file.

Supplemental Table 1. Primer sequences for real-time PCRs.

	Forward primer sequence (5'-3')	Reverse primer sequence (5'-3')
**Mouse *TNF***	AACTCCAGGCGGTGCCTAT	TGCCACAAGCAGGAATGAGA
**Mouse *TGFβ1***	CCACCTGCAAGACCATCGAC	CTGGCGAGCCTTAGTTTGGAC
**Mouse *IL-17A***	TGACGCCCACCTACAACATC	CATCATGCAGTTCCGTCAGC
**Mouse *CCL20***	GCCTCTCGTACATACAGACGC	CCAGTTCTGCTTTGGATCAGC
**Mouse *IL-1β***	TTCAGGCAGGCAGTATCACTC	GAAGGTCCACGGGAAAGACAC
**Mouse *IL-6***	TAGTCCTTCCTACCCCAATTTCC	TTGGTCCTTAGCCACTCCTTC
**18s ribosomal RNA**	ATTCGAACGTCTGCCCTATCA	GTCACCCGTGGTCACCATG
**Total bacterial 16S rRNA**	515F (GTGCCAGCMGCCGCGGTAA)	806R (GGACTACHVGGGTWTCTAAT)
**SFB 16S rRNA**	736F (GACGCTGAGGCATGAGAGCAT)	844R (GACGGCACGGATTGTTATTCA)
